# Radial Head Dislocation with Elbow Subluxation in an Adult

**DOI:** 10.7759/cureus.5570

**Published:** 2019-09-05

**Authors:** Amanda L Webb, Mary C Slome, Ayanna Walker, Latha Ganti

**Affiliations:** 1 Emergency Medicine, University of Central Florida College of Medicine / Hospital Corporation of America Graduate Medical Education Consortium of Greater Orlando, Orlando, USA; 2 Emergency Medicine, Envision Physician Services, Orlando, USA

**Keywords:** radial head dislocation, elbow subluxation

## Abstract

Isolated radial head subluxation without fracture, commonly referred to as “nursemaid’s elbow,” is one of the most common pediatric upper extremity injuries. Radial head dislocation without an associated fracture is rarely seen in adults. They are usually associated with ulnar fractures or an elbow dislocation. We present a case of an adult female presenting with a radial head dislocation and an elbow subluxation sustained while dressing, which was successfully reduced using the techniques commonly used to reduce nursemaid’s elbow in pediatric patients.

## Introduction

Isolated radial head subluxation, also referred to as “nursemaid’s elbow” or “pulled elbow,” is the most common upper extremity injury in children younger than six years old, peaking at two to three years [1-3]. Roughly half to two-thirds of the cases are caused by the classical mechanism of axial traction applied to the forearm (pulling), allowing the annular ligament to slip and become entrapped between the radial head and capitellum [2-6]. Radial head subluxations or dislocations rarely present without an associated fracture or elbow dislocation in adults [7-9].

## Case presentation

A 43-year-old female with a self-reported history of prior elbow dislocations presented to the emergency department with pain, deformity, and decreased range of movement in her elbow after dislocating her elbow while dressing. Unlike her prior dislocations, she was unable to reduce it herself. She was unable to provide further details about her prior elbow injuries.

**Figure 1:**
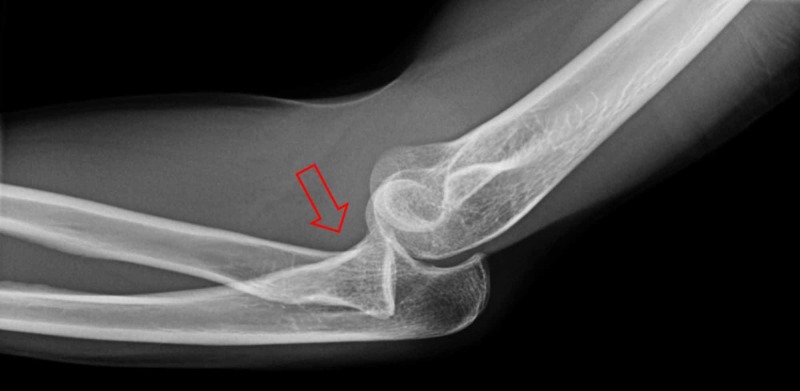
Pre-reduction lateral X-ray of the elbow showing a distal radial head dislocation with elbow subluxation

Physical exam revealed deformity to the left elbow with a decreased range of motion. Radial pulse was 2+ and sensation was intact distally. There was no swelling, radial head tenderness, erythema, or warmth present. Radiograph of the elbow demonstrated a dislocation of the radial head and a subluxed elbow joint without obvious fracture (Figure 1).

**Figure 2:**
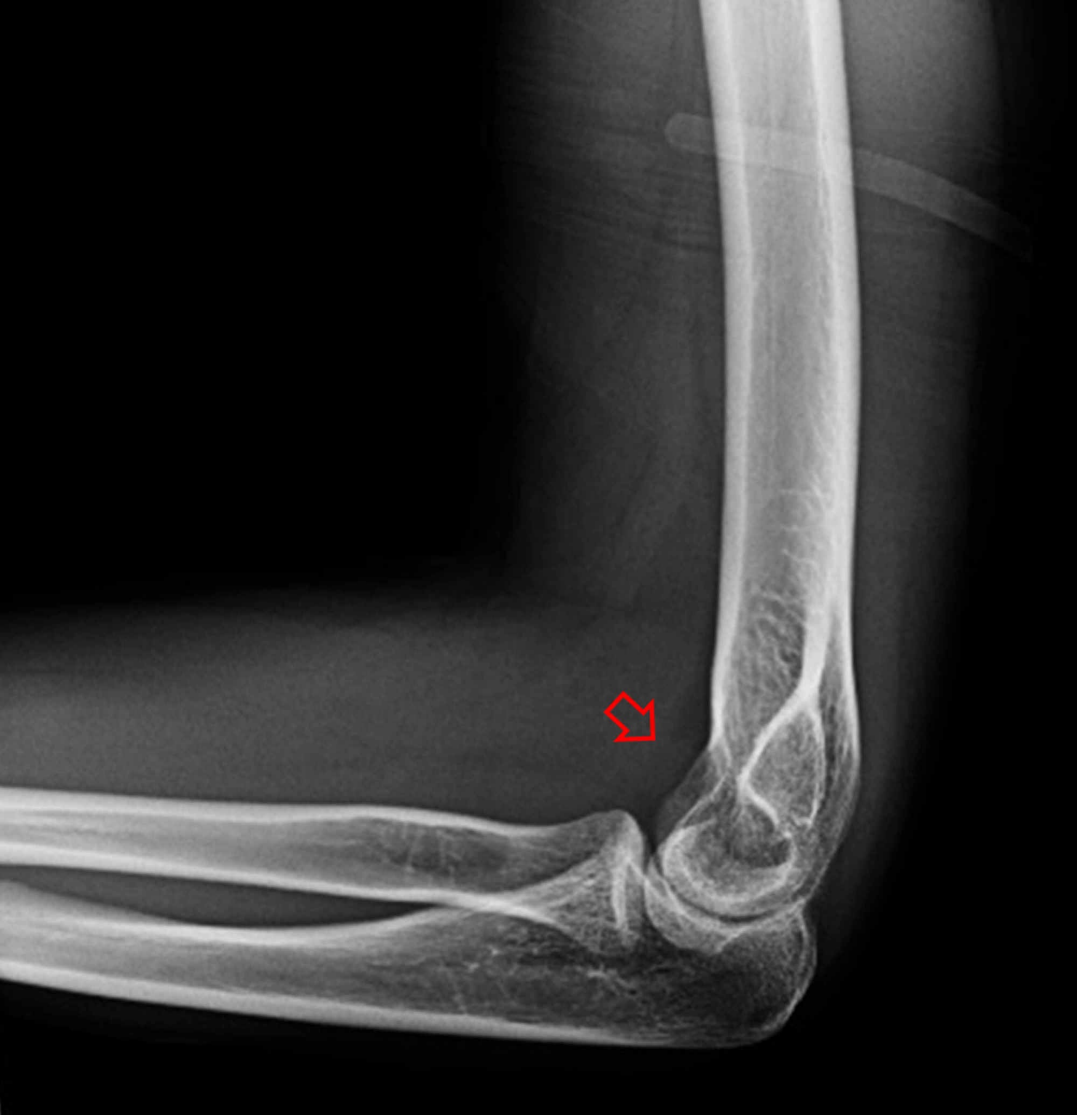
Post-reduction lateral X-ray of the elbow showing reduction of the radial head and elbow joint. Suspected hemarthrosis is indicated by the arrow.

Etomidate was used for sedation in small aliquots, to a total of 10 mg, for procedural sedation. The joint was reduced by hyperpronation followed by supination with flexion while applying pressure on the radial head. She tolerated the procedure well and without complication. Pain and range of motion improved post-reduction. Post-reduction films showed a suspected hemarthrosis in addition to the resolution of the dislocation and subluxation (Figure 2). She was placed in a sling with swathe and was discharged with intact neurovascular function with orthopedic follow-up.

## Discussion

Although radial head subluxation is common in children under the age of six, isolated radial head dislocation without an associated fracture or elbow dislocation is rare, and even more so in older children and adults [7-9]. Posterior radial head dislocations are less common than anterior ones [9]. As children age, the annular ligament becomes thicker and has stronger bony attachments [1]. Although the annular ligament is described as being the primary stabilizer of the proximal radioulnar joint and is associated with radial head subluxation, other structures also play a crucial role. The quadrate ligament aids the annular ligament in preventing posterior displacement of the radial head [9]. The typical presentation for a radial head dislocation is the inability to supinate and pronate while still being able to flex and extend [10]. Anterior and anterolateral radial head dislocations have been described after falling and twisting a hyperextended elbow and from a motorbike accident [11-12].

Radial head subluxations are easily reduced with as high as a 98% success rate using hyperpronation [1]. Supination and flexion are also used but with a slightly lower success rate [1,10]. The majority of cases reported using pronation to reduce the radial head followed by immobilization in the flexed and pronated position, although others note success reducing using supination and immobilizing in the flexed and supinated position [8,11-15]. It has been suggested that posterior radial head displacement may be prevented during pronation by the bony contact when the ulna and the radius cross [9].

Functional outcome is generally good if reduced in the acute setting. However, missed diagnosis or delay in treatment requires surgical intervention and can lead to poor functional outcome, degenerative arthritis, elbow joint instability, and/or reduced elbow flexion [8,12,16-17]. These injuries can be easily missed, especially in cases with polytrauma, as elbow flexion and extension have little to no impairment [8,18-19]. The elbow should be assessed during pronation and supination for pain and reduced range of motion. The radiocapitellar joint alignment on radiographs can be assessed by drawing a line through the middle of the shaft longitudinally. The line should intersect the capitellum [16].

## Conclusions

Radial head dislocations in adults without an associated fracture or elbow dislocation are rare but are generally amenable to closed reduction in the emergency department. They can be easily missed on exam, as there is generally minimal to no effect on elbow flexion and extension. We recommend testing pronation and supination on exam to avoid missing this injury. Delay in care can lead to the need for surgical intervention.
